# Cost of Military Patients in Civil Hospitals

**Published:** 1918-06-01

**Authors:** 


### COST OF MILITARY PATIENTS IN CIVIL HOSPITALS.

### The Inquiry Upon Which the Present Grant was Based.

WHEN a saint is canonised some person is told off to act as "the Devil's Advocate." It is his duty to bring forward every reason that can occur to him, after examination of the evidence, against the proposed posthumous honour.

When the British Hospitals Association, acting on behalf of those voluntary hospitals that take military patients into their wards, applied to the War Office for an increase in the capitation grant for such patients, on the plea that since the grant was last fixed the cost of maintenance had considerably risen, the War Office appointed two gentlemen—Dr. E. Napier Burnett and Mr. R. Orde—to examine the evidence in support of the claim. These gentlemen necessarily found themselves in the nature of things very much in the position of the Devil's Advocate; it was their mission to find evidence against canonisation, and it says something for the manner in which the case of the hospitals was prepared by the British Hospitals Association, and supported by the hospital officers who were selected as witnesses, that it was ultimately decided to increase the grant from 4s. to 4s. 9d. per patient per day.

The report of the representatives of the War Office has now been printed and circulated by the British Hospitals Association amongst the hospitals concerned. Let it be said at once that it is an extremely interesting and able document, distinguished by a human tone of argument and phraseology almost invariably absent from a report issued by a Government Department. If it contains, on the side of suggestion, little that is not known, or should be known, to the skilled hospital administrator, it should do much to help to revive methods and refresh memory in respect of phases of administrative work which in, we fear, too many instances have been allowed to be relaxed during the strain of the last few years.

### THE METHOD OF INQUIRY.

This said, let us examine, in as much detail as space will permit, the findings of the Government investigators and the steps by which they arrived at their conclusions. After consultation with the Rev. G. B. Cronshaw, of the Radcliffe Hospital, and Mr. E. W. Morris, house governor of the London Hospital, sixteen hospitals of a more or less representative type were selected and visited. In each locality a hospital with a medical school and a hospital without were chosen, and in advance of the visit a list of questions was sent to the secretaries. These questions had to do with the numbers of patients and staff fed in the hospital during a given period, and were supplemented, on the arrival of the representatives, by other questions as to numbers of the staff and their remuneration and divers other matters. In this way a mass of information was collected, part of which is utilised in the report and part has not yet been classified, but all of which, the report states, goes to support the conclusions set out in the recommendations.

The report expresses regret that the present system of hospital accounts does not allow of the separation of the cost of food consumed by the patients from that provided for the staff. In the absence of this information (which, in passing, we venture to state should have been available in every up-to-date institution), and having regard to the fact that the food figures were for 1916 and the staff figures were for 1917, the investigators have approximated the average cost per patient, for food, at 20.7d. per day. The data revealed a remarkable level—from 19.5d. to 21.5d. The report agrees that these figures are for 1916, and that the claim is based on the undoubted fact that prices have considerably risen since that year.

### DO SOLDIERS NEED FEWER NURSES?

But counterbalancing factors are urged. It is pointed out that on the whole the military patients need less nursing than the civilian patients, and, as a matter of fact, fewer nurses were found on the military side in most instances. Here we should like to quote an expression of opinion which is peculiarly appropriate to counsel for the defence. "In two hospitals we were informed that there is no reduction in the number of nursing staff for military patients as compared with civil. In peace times it is all very well to live up to ideals and maintain an ideal standard of nursing, but to-day, as long as the interests of the military patients are reasonably safeguarded, we cannot take the view that in the circumstances economies should be effected in every other department of a hospital and that nursing should be maintained at a peace-time standard."

Yes, but a hospital that is a training-school for nurses, either by itself or in conjunction with another hospital, cannot make and change its scheme of nursing on the basis of the medical nature of some of the cases having temporarily changed. Its scheme would often be utterly upset and all continuity lost if it discharged or re-engaged nurses at the end of comparatively short periods. Moreover, those whose duties include the presentation of statistics of cost would be confronted by the nightmare of a differential calculation in respect of the cost of a patient with a broken leg and of one acutely ill with pneumonia.

### A CASE OF TOO AMPLE FEEDING.

But let us resume. It is admitted that the military patient costs more to feed than the civilian. The estimates of secretaries varied from an increase in cost of 10 per cent. to one of 50 per cent. In one hospital a scale of feeding had been required by the Inspecting General so liberal that the patients were unable to eat the amount supplied. But against this the lack of foodstuffs had resulted in substantial economies so far as staff is concerned, and, generally, important reductions had been enforced by circumstances. In one hospital the milk had been reduced by one-third, elsewhere "extras" had been cut down to vanishing point, nearly everywhere sugar had been reduced and margarine was being used instead of butter, but the report does not mention the probability that these economies refer to quantities used, and not to gross cost as compared between 1916 and late in 1917. This fact is brought out by the interesting tables of prices presented. In one hospital milk had increased in price from 1s. 1d. to 2s. 1d. per gallon, sugar from 43s. 3d. to 49s. per cwt., and margarine from 91s. 4d. to 112s. per cwt. It is interesting to learn that London is not in a worse position to buy milk than any other part of the country. In respect of the part of the report dealing with food it is concluded that fewer nurses, rationing of food, use of substitutes, reduction of extras, attention to cooking and serving of food, and deduction in respect of the cost of out-patients, taken in conjunction, go far to discount the undoubted rise in prices which has taken place during 1917.

### COST OF MILITARY PATIENTS—(*continued*).

In dealing with the department of Surgery and Dispensary, the excellent advice is given that more use should be made of the comparative statement. The sister and the surgeon are quite susceptible to the lessons gained from comparing the cost of their work with that of officers doing similar work in other wards or operating theatres. "Here and there a crank may refuse to submit to the control of a comparative return, or may even be driven to greater extravagance, but cranks are in the minority, and the surgeons in military and civil hospitals are as amenable to reason as other men." We wish we could quote at length from this part of the report, which is bristling with excellent suggestions and information. We read, as an instance, how in one hospital, when attention had been given to the matter, the consumption of methylated spirit had been reduced from 1,500 gallons in the year to 150, and that whereas formerly one surgeon used spirit in a basin he now used it in a cup.

It is truly said that the dispensary of a hospital is one of its most important departments. A practical and capable dispenser with a properly equipped laboratory is able to ensure that the stuff bought is up to the standard and worth the money paid for it. To take one instance, that of soap. It is well known that soap can contain almost any percentage of water, and without analysis a hospital may be paying what it imagines to be a low price, but is in reality a high price, for the actual soap the mixture contains, and quite a considerable balance of water.

### THE VALUATION OF STOCKS.

The report brings out the important point of stocks. King Edward's Hospital Fund do not require the showing of stocks in the accounts; their view is that they wish to discourage hospitals holding stocks. Yet there is no doubt that many hospitals have bought largely ahead, especially in articles coming under Surgery and Dispensary. In one case it is known that pre-war stuff is still being used.

Under Domestic, laundry, coal, gas, and water are chiefly selected for comment, and many interesting tables are presented revealing remarkable deviation in cost. A useful truism in respect of washing is brought out. It is not generally realised that the life of a sheet or garment is in so many washings. Every time, therefore, an article goes needlessly to the wash not only is its life shortened unnecessarily, but the fact is hidden under cost per article. The more articles needlessly washed, the greater the divisor and the lower the cost of washing per article.

Secretaries of hospitals having their own laundries will do well to read with great attention the considerable part of the report dealing with the laundry. The question is in the aggregate important from the point of view of national finance. If the daily average occupation of military hospitals is, say, 200,000, and if we take the average weekly washing of each of the patients as one article above the necessary, we reach the enormous total of 10,400,000 pieces washed unnecessarily each year. If the life of an article is 100 washings, there is an annual unnecessary destruction of 104,000 pieces. This destruction is actually *paid* for to the tune of £43,000 per annum, calculating washing at 1d. per article.

### NURSES' HOURS TOO LONG.

There are useful tables showing average cost under salaries and wages, nurses' hours of duty, and the numbers employed in the kitchens of the hospitals chosen for illustration. The Devil's Advocate is at times on the side of the angels; the opinion is expressed that "we are strongly in sympathy with the efforts now being made by a few hospitals to secure not only better rates of pay and a more efficient training for nurses, but also to obtain that no nurse shall be on ward duty for more than eight to nine hours a day. The quality of a nurse's work is so important that we believe no nurse is capable of doing the best for her patients when obliged to work long hours."

To summarise the recommendations, 1s. 8d. is considered to be sufficient to represent the daily cost of a patient under food (not necessarily the cost as it is, but the cost as it should be), 4d. for drugs and dressings, 1s. for domestic expenses, and 1s. 4d. for salaries and wages. The remaining 5d., to make up a 4s. 9d. grant, is presumably to cover the cost of establishment, miscellaneous expenses, administration, and rent, rates, and taxes, in respect of all of which charges the report is discreetly silent.

It is cold comfort to the hospital with an average cost of 7s. a day per bed (a figure not unusual in these times) to read this report; but this must be said about the matter, that any hospital which studies with care the suggestions given should be able to reduce cost appreciably. There is hardly a method advocated in this able document which we have not urged, in these columns and elsewhere, over a long period of years, but it is little to us if a new prophet has arisen with an old prophecy, should a useful system of checks on expenditure be revived where it has been discontinued, or inaugurated where it has never been commenced.

